# How to properly define immunological nonresponse to antiretroviral therapy in people living with HIV? an integrative review

**DOI:** 10.3389/fimmu.2025.1535565

**Published:** 2025-04-07

**Authors:** Maria Carolina Santos Guedes, Henrique Fernando Lopes-Araujo, Kleyverson Feliciano dos Santos, Esaú Simões, Wlisses Henrique Veloso Carvalho-Silva, Rafael Lima Guimarães

**Affiliations:** ^1^ Department of Genetics, Federal University of Pernambuco (UFPE), Recife, Pernambuco, Brazil; ^2^ Keizo Asami Institute (iLIKA), Federal University of Pernambuco (UFPE), Recife, Pernambuco, Brazil; ^3^ Department of Immunology, Aggeu Magalhães Institute (IAM/FIOCRUZ), Recife, Pernambuco, Brazil; ^4^ Life Sciences Nucleus, Agreste Academic Center (CAA), Federal University of Pernambuco (UFPE), Caruaru, Pernambuco, Brazil

**Keywords:** AIDS, ART, immunological non-responders, CD4+ T-cell reconstitution, immunological classification

## Abstract

In recent decades, significant progress has been made in understanding the mechanisms underlying human immunodeficiency virus (HIV) infection and its treatment. Antiretroviral therapy (ART) has notable improved the life expectancy and quality of life for people living with HIV (PLHIV) by suppressing viral replication and promoting CD4+ T-cell recovery. However, despite its efficacy, approximately 10-40% of ART-treated PLHIV with virological suppression (<50 RNA copies/mL) do not achieve adequate immunological reconstitution. These PLHIV, classified as immunological non-responders (INR), experience higher morbidity and mortality rates compared to those with satisfactory immune reconstitution, known as immunological responders (IR). Various studies have explored the mechanisms contributing to immunological nonresponse, yet a major challenge remains: the lack of a standardized definition of immunological response and nonresponse across studies. Currently, definitions are inconsistent, limiting comparability between studies. This review proposes a clear and adequate classification for IR and INR PLHIV to support future advancements in understanding immunological recovery and improving the quality of life for ART-treated PLHIV.

## Introduction

Since the identification of human immunodeficiency virus (HIV) as the causative agent of acquired immunodeficiency syndrome (AIDS), significant progress has been made in treatment of people living with HIV (PLHIV) (1). Advances in understanding the virus’s pathogenesis have enabled the development of new drugs and therapeutic strategies (2, 3). Consequently, many PLHIV have experienced an increase in life expectancy, with HIV infection now being managed as a chronic condition rather than a life-threatening disease (4). As a result, the life expectancy of PLHIV has increased to over 50 years (5). Currently, global data indicate that approximately 29 million PLHIV (76% of all PLHIV) are on antiretroviral therapy (ART), and 93% of them have achieved a reduction in plasma viral load to undetectable levels (<50 RNA copies/mL) (6).

Despite substantial advancements in understanding HIV infection, certain aspects such as the immune reconstitution of PLHIV on ART remain unclear (1). Normally, reducing viremia leads to a gradual recovery of CD4+ T-cell count over time (7). However, 10-40% of ART-treated PLHIV experience impaired immune reconstitution, characterized by limited CD4+ T-cell recovery even after virological suppression (8, 9). This condition is associated with an increased risk of HIV-related complications and death, and these PLHIV are defined as virological responders but immunological non-responders (INR) (9).

Several studies have suggested various mechanisms to explain the deficiency in immune reconstitution among ART-treated PLHIV (8–10). However, the lack of consensus among researchers on defining criteria for classifying immunological nonresponse has become a significant obstacle to fully understanding this condition. Thus, the present study aimed to propose a classification to define immunological nonresponse in ART-treated PLHIV, establishing a fundamental framework for future studies focused on elucidating the mechanisms involved in unsatisfactory immune reconstitution.

## Immunological recovery in ART-treated PLHIV

In the first decade following the development of ART, there were different indications for its initiation, particularly regarding CD4+ T-cell count (11). Primarily, the World Health Organization (WHO) recommended initiating treatment in PLHIV with a CD4+ T-cell count ≤500 cells/mm^3^ (12, 13). However, in the early 2000s, the criteria were revised to a threshold of ≤200 cells/mm^3^ due to concerns about antiretrovirals toxicity (13). In 2006, the WHO adjusted its recommendation to initiate treatment in PLHIV with a CD4+ T-cell count of 350 cells/mm^3^, which was later revised back to ≤500 cells/mm^3^ (14). Currently, studies have demonstrated that early initiation of ART, regardless of CD4+ T-cell count, leads to a more rapid reduction in viral load, better immune reconstitution, and decrease in mortality (15). Thus, ART is now recommended to be initiated within 7 days after diagnosis, following the “treat-all” approach established by the WHO in 2017 (16, 17).

**Figure 1:**
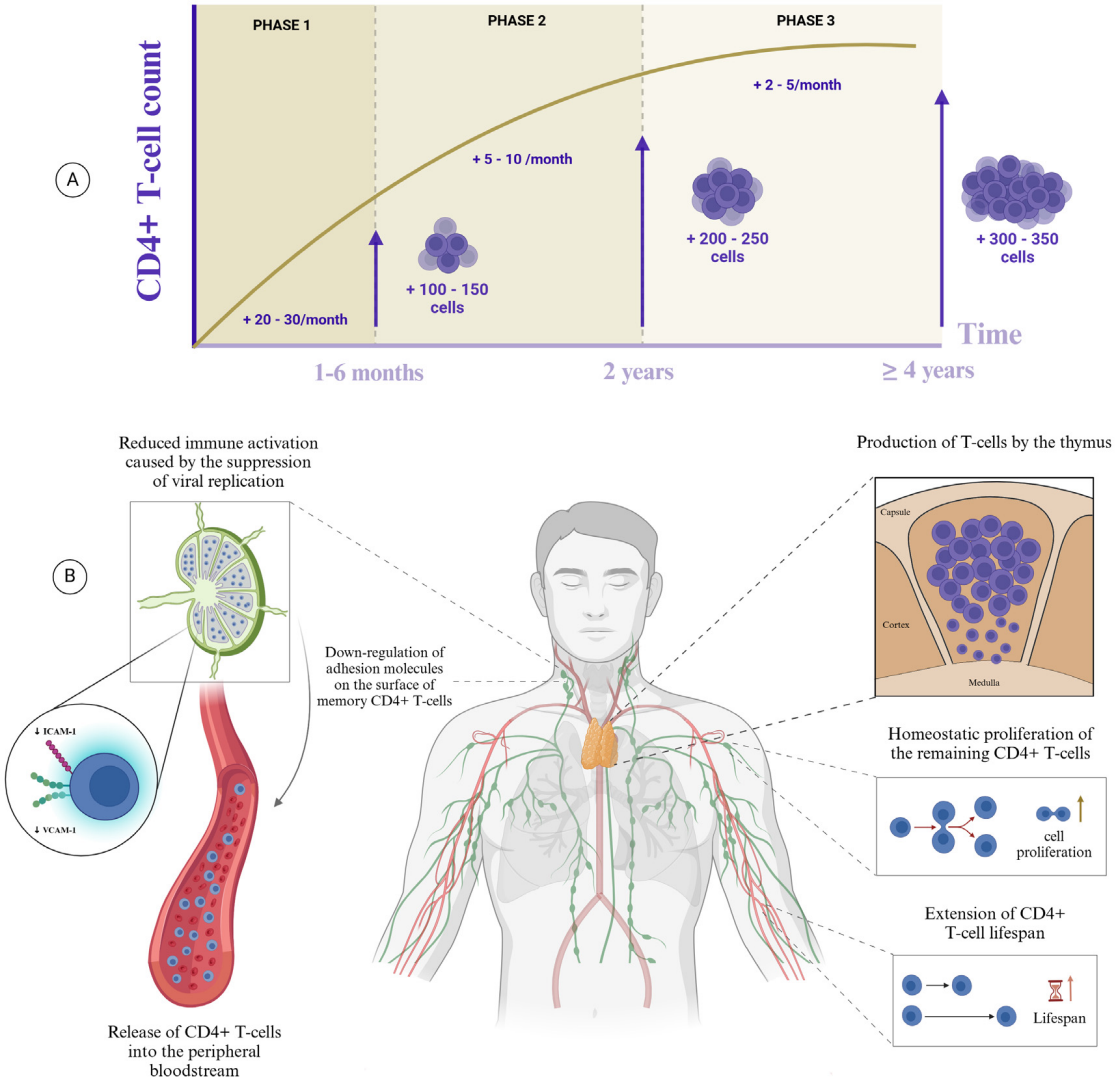
Immunological recovery in PLHIV during ART. **(A)** Average CD4+ T-cell count reconstitution in ART-treated PLHIV. CD4+ T-cell count reconstitution in PLHIV undergoing ART is divided into three phases, each with distinct rates of cell gain. In the first phase, spanning 1 to 6 months of ART, CD4+ T-cell counts increase by approximately 20-30 cells/µL per month. The second phase, from 6 months to the second year of ART, shows a gain of 5-10 cells/µL per month. The third phase, extending beyond the second year and for at least 7 years, is characterized by a slower increase of 2-5 cells/µL per month. **(B)** Mechanisms responsible for CD4+ T-cell count increase during ART. The initial increase in CD4+ T-cell count following ART initiation results from reduced immune activation, allowing memory CD4+ T-cells to migrate from lymphoid organs into the peripheral circulation. Over time, additional mechanisms – such as thymic production, peripheral proliferation of CD4+ T-cells, and prolonged cell lifespan – support the gradual rise in T-lymphocytes throughout treatment.

Successful therapy is expected to be followed by a gradual restoration of immunological function (**Figure 1A**) (18). Initially, treatment reduces viremia, which in turn decreases immune activation induced by HIV infection (19). In most PLHIV, plasma viral load reaches undetectable levels within six months (20, 21). This reduction downregulates the expression of intercellular adhesion molecule-1 (ICAM-1) and vascular cell adhesion molecule-1 (VCAM-1) on the surface of CD4+ T lymphocytes, the primary molecules responsible for retaining these cells within lymphoid organs (22). As a result, memory CD4+ T-cells are redistributed from lymphoid tissues to peripheral blood, increasing the circulating T-cell count (**Figure 1B**). This redistribution characterizes the initial phase of immunological recovery, usually resulting in a gain of 20-30 cells/µL per month and lasts for approximately 6 months (**Figure 1A**) (7).

During the second phase, CD4+ T-cell reconstitution is predominantly driven by thymic production, with an average gain of 5-10 cells/µL per month (7, 23). In this phase, larger thymic size is associated with higher percentages of T-cell receptor excision circles (TRECs), recent thymic emigrants (RTEs) and naïve CD4+ T-cell (23). By the end of the second year of ART, PLHIV typically exhibit a gain of approximately 200 cells/µL (**Figure 1A**) (7, 24). During this period, CD4+ T-cells also gradually begin to recover their antigen-specific response to various antigens, except HIV (9). Notably, the first two years of ART represent the most significant increase in cell count, with subsequent years showing minimal gains. Some studies suggest a tendency to initiate a plateau in CD4+ T-cell recovery after this period (23, 25, 26).

In addition, two other mechanisms contribute during the second and third phases: the homeostatic proliferation of residual CD4+ T-cells and an increased lifespan of these cells (**Figure 1B**) (27). The third phase, spanning from the third to the seventh year of ART, is responsible for a gain of 2-5 cells/µL per month (**Figure 1A**) (7). Although these mechanisms contribute to the increase in CD4+ T-cell count, they are not qualitatively equivalent. Only thymic production has the potential to partially restore the patient’s antigenic repertoire (7).

Although most processes involved in immune reconstitution have been described, ongoing debate remains regarding the optimal CD4+ T-cell count threshold indicative of therapeutic immunological success in PLHIV under ART. In healthy individuals not infected with HIV, the typical CD4+ T-cell count ranges between 500 and 4000 cells/µL (28). Consequently, many studies have reached a consensus that PLHIV treated with ART who achieve a CD4+ T-cell count above 500 cells/µL exhibit morbidity and mortality rates like those of HIV-negative individuals (29). At this cell count level, these PLHIV demonstrate reduced susceptibility to non-AIDS events and are considered to have restored immunocompetence (8, 30–32).

Understanding the immunological response capacity of PLHIV is essential, as it has been demonstrated that different CD4+ T-cell counts can affect their susceptibility to various coinfections and neoplasms (33). For instance, PLHIV with a CD4+ T-cell count below 100 cells/µL (in AIDS stage) are more susceptible to developing esophageal candidiasis, toxoplasmic encephalitis, and primary central nervous system lymphoma (PCNSL) (33). Meanwhile, in PLHIV with 200-500 cells/µL, conditions such as oral hairy leukoplakia, mucocutaneous Kaposi sarcoma, and cervical or anal neoplasia are more common (34). In contrast, ART-treated PLHIV with a CD4+ T-cell count above 500 cells/µL manifest similar infections to those seen in HIV-negative individuals (33). Hence, this threshold has garnered widespread acceptance as a marker of satisfactory immune reconstitution in PLHIV undergoing ART (8). Conversely, inadequate CD4+ T-cell gain directly impacts the infection’s prognosis and indicates immunological nonresponse (35).

## Immunological nonresponse to ART

**Figure 2:**
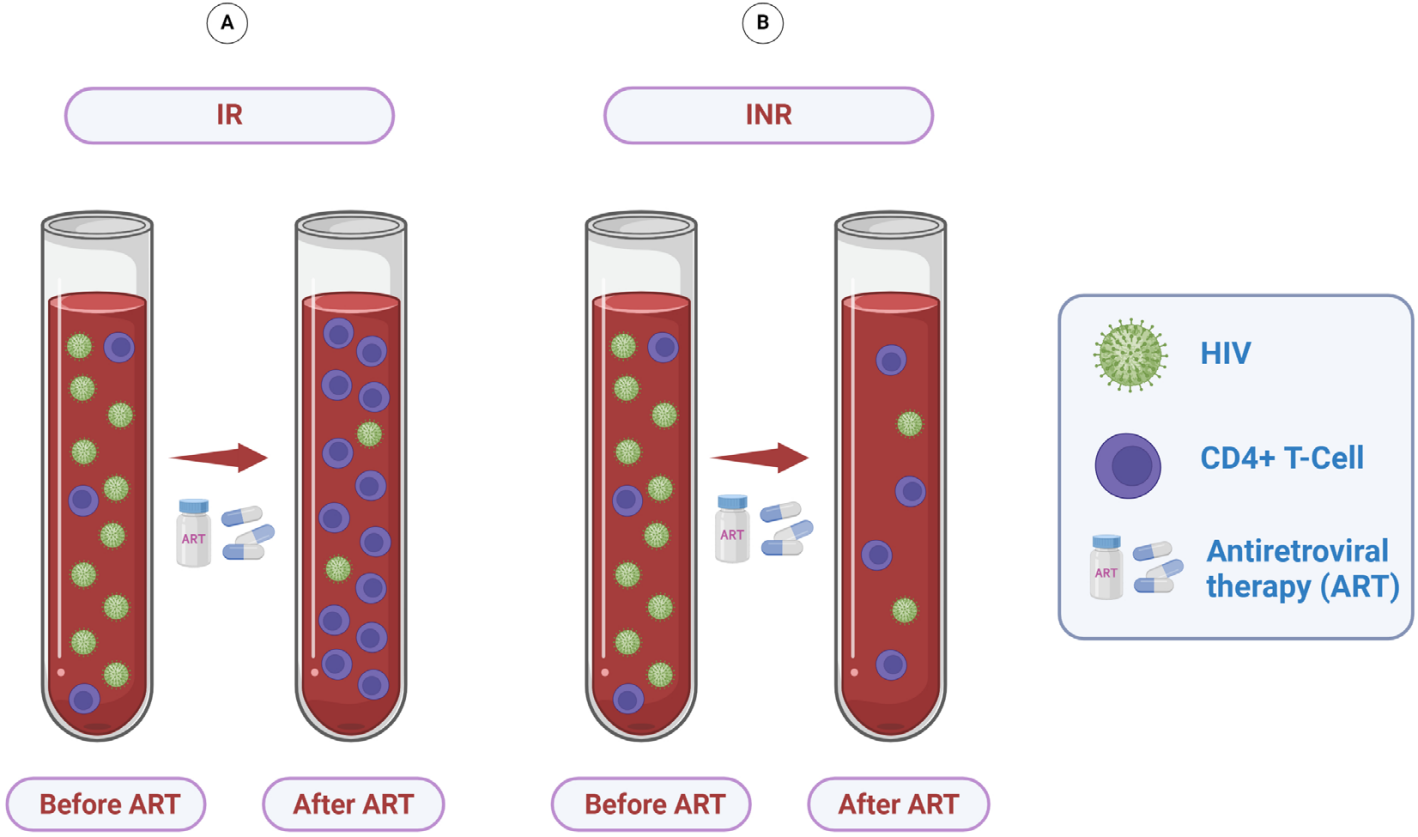
Immunological response to ART. Following the initiation of ART, plasma viral load markedly decreases to undetectable levels, creating conditions favorable for CD4+ T-cell recovery. **(A)** PLHIV who experience satisfactory immune reconstitution are defined as immunological responders (IR). **(B)** Despite achieving virological success, some PLHIV exhibit reduced CD4+ T-cell reconstitution and are thus classified as immunological non-responders (INR).

Therapeutic success in ART-treated PLHIV has traditionally focused on suppressing viral load. Nevertheless, it can also be characterized by distinct stages of CD4+ T-cell recovery (36). Consequently, the objective of ART has expanded beyond merely controlling viral replication to foster an environment conducive to immune reconstitution (10). However, a significant portion of ART-treated PLHIV, ranging from 10% to 40%, exhibit persistently low CD4+ T-cell counts despite achieving virological suppression, categorizing them as immunological non-responders (INR, **Figure 2B**) (37). In contrast, PLHIV who successfully achieve immune reconstitution are classified as immunological responders (IR, **Figure 2A**) (8). Approximately two decades ago, initial studies first identified this condition (38, 39), emerging two years after the introduction of combination therapy for treating PLHIV (40). Since then, further research has provided new insights into the complexities of immunological nonresponse (8, 10, 41)

Immunological nonresponse in PLHIV presents significant risks for infection prognosis, including progression to AIDS-defining events and increased mortality (42). Studies have shown that the mortality rate is 100 times higher in PLHIV with a CD4+ T-cell count below 50 cells/µL compared to those with a count above 500 cells/µL (30). Moreover, the immune dysfunction observed in INRs is also associated with the development of non-AIDS events, such as metabolic syndrome, cardiovascular diseases, and nephropathy (43, 44).

**Figure 3:**
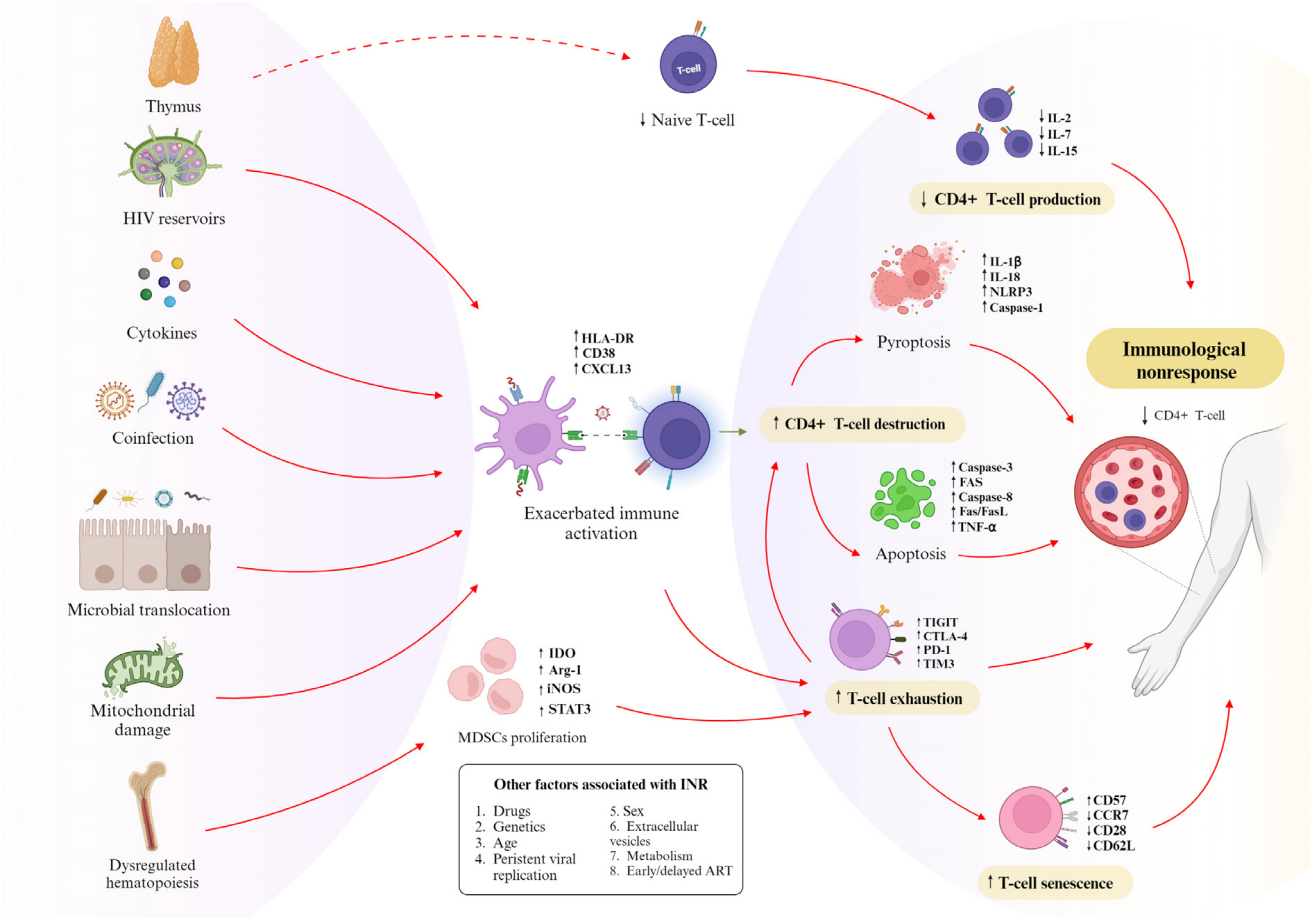
Factors related to immunological nonresponse. The main factors associated with immunological nonresponse include HIV reservoirs, cytokine dysregulation, coinfections, microbial translocation, mitochondrial damage, and dysregulated hematopoiesis. These factors can lead to exacerbated immune activation and increased proliferation of MDSCs, which contribute to increased CD4+ T-cell exhaustion and destruction, resulting in immunological nonresponse in ART-treated PLHIV. Reduced thymic output can contribute to decreased CD4+ T-cell production, which also result in immunological nonresponse Additionally, other factors such as drugs effects, genetic alterations, older age, persistent viral replication, male sex, extracellular vesicles, metabolic conditions, and late ART initiation may also be associated with the INR group. ART, antiretroviral therapy; INR, immunological non-responders; MDSCs, myeloid-derived suppressor cells; PLHIV, people living with human immunodeficiency virus.

Immunological nonresponse is considered a multifactorial condition, with several factors identified as risk contributors, including male sex, advanced age, coinfections, exacerbated immune activation, thymic exhaustion, genetic alterations, and others (**Figure 3**) (45, 46). Additionally, reduced T-lymphocytes production and increased destruction of these cells are recognized as the primary processes associated with immunological nonresponse, given that T-cell homeostasis is crucial for effective immune reconstitution (47). Previous studies have demonstrated that INRs experience a reduction in the output of recent thymic emigrants (RTE), indicating thymic insufficiency, with these cells exhibiting an increased rate of cell death via pyroptosis – one of the main types of cell death observed in PLHIV (48, 49). Other immune dysfunctions observed in INRs include an altered cytokine secretion profile, disruptions in regulatory components such as T-reg and Th17 cells, mitochondrial dysfunction and dysregulated hematopoiesis, which results in the abnormal proliferation of myeloid-derived suppressor cells (MDSCs), T-cell exhaustion and senescence (50).

Another crucial element influencing immune reconstitution is the pre-ART CD4+ T-cell count (51). Previous research conducted by our research group revealed that most INRs had a CD4+ T-cell count of ≤200 cells/µL at the initiation of ART (52, 53). Furthermore, it has been suggested that starting ART with a baseline CD4+ T-cell count of ≥500 cells/µL significantly enhances the likelihood of satisfactory immunological recovery (15). This underscores the importance of initiating ART regardless of CD4+ T-cell count, as PLHIV who delayed treatment until their count dropped below 200 cells/µL did not achieve immune reconstitution even after 10 years of viral suppression (54). This issue is particularly pronounced in older PLHIV (55). Additionally, a low pre-ART CD4+ T-cell count is associated with the emergence of X4 strains, co-infections, and microbial translocation (9, 56). Other contributors to immunological nonresponse include persistent residual viral replication within reservoirs, issues related to drug efficacy and delayed initiation of ART (41)

## Classifications of immunological responders and non-responders

Although studies on immunological recovery are increasingly abundant, there remains no consensus among authors on the definition of immunological nonresponse. Criteria such as absolute CD4+ T-cell count, CD4+ T-cell increase, CD4/CD8 ratio, or combinations of them have been adopted in various classifications (**Table 1**). Furthermore, differences in the time frames required for these classifications and other factors contribute to a lack of standardization (41, 120). This diversity highlights the need to compile these classifications and recommend a more standardized approach.

**Table 1:** Definitions of immunological responders and immunological non-responders.

Criteria	Immunological Responders (IR)	Immunological Non-Responders (INR)	References
Absolute CD4+ T-cell count	–	250 cells/µL following clinical failure or a persistent count of 100 cells/µL after six months of effective treatment	(16)
	> 500 cells/μL from baseline at 5 years after ART initiation	< 500 cells/μL from baseline at 5 years after ART initiation	(57, 58)
	> 500 cells/μL from baseline at 2-4 years after ART initiation	< 500 cells/μL from baseline at 2-4 years after ART initiation	(59, 60)
	> 500 cells/μL from baseline at 1 year after ART initiation	< 500 cells/μL from baseline at 1 year after ART initiation	(61)
	> 600 cells/μL from baseline at 2 years after ART initiation	< 400 cells/μL from baseline at 2 years after ART initiation	(62)
	> 500 cells/μL from baseline at 1 year after ART initiation	< 400 cells/μL from baseline at 1 year after ART initiation	(63)
	≥ 500 cells/μL from baseline at 2-3 years after ART initiation	≤ 350 cells/μL from baseline at 2-3 years after ART initiation	(64–70)
	> 400 cells/μL from baseline at 2 years after ART initiation	< 350 cells/μL from baseline at 2 years after ART initiation	(71)
	> 350 cells/μL from baseline at 8 years after ART initiation	< 350 cells/μL from baseline at 8 years after ART initiation	(72)
	≥ 350 cells/μL from baseline at 4 years after ART initiation	< 350 cells/μL from baseline at 4 years after ART initiation	(73)
	> 350 cells/μL from baseline at 1-2 years after ART initiation	≤ 350 cells/μL from baseline at 1-2 years after ART initiation	(35, 74–83)
	–	< 350 cells/μL from baseline at 1 year after ART initiation	(84)
	–	≤ 350 cells/μL from baseline at 24-36 weeks after ART initiation	(85, 86)
	> 270 cells/μL from baseline at 96 weeks after ART initiation	< 270 cells/μL from baseline at 96 weeks after ART initiation	(87)
	≥ 500 cells/μL from baseline at 48 weeks after ART initiation	≤ 250 cells/μL from baseline at 48 weeks after ART initiation	(88)
	> 250 cells/μL from baseline at 3 years after ART initiation	< 250 cells/μL from baseline at 3 years after ART initiation	(18)
	–	< 250 cells/μL from baseline at 1-2 years after ART initiation	(89, 90)
	> 250 cells/μL from baseline at 2 years after ART initiation	< 200 cells/μL from baseline at 2 years after ART initiation	(91)
	≥ 200 cells/μL from baseline at 3 years after ART initiation	< 200 cells/μL from baseline at 3 years after ART initiation	(92)
	≥ 350 cells/μL from baseline at 1 year after ART initiation	≤ 200 cells/μL from baseline at 1 year after ART initiation	(93)
	≥ 500 cells/μL from baseline at 2 years after ART initiation	≤ 200 cells/μL from baseline at 2 years after ART initiation	(37, 94–96)
	–	< 200 cells/μL from baseline at 6 months after ART initiation	(97)
CD4+ T-cell count increase	> 400 cells/μL from baseline at 5 years after ART initiation	< 400 cells/μL from baseline at 5 years after ART initiation	(98)
	≥ 200 cells/μL from baseline at 1-2 years after ART initiation	< 200 cells/μL from baseline at 1-2 years after ART initiation	(99, 100)
	> 100 cells/μL from baseline at 1 year after ART initiation	< 100 cells/μL from baseline at 1 year after ART initiation	(101, 102)
	> 100 cells/μL from baseline at 48 weeks after ART initiation	< 100 cells/μL from baseline at 48 weeks after ART initiation	(103)
	> 100 cells/μL from baseline at 1 year after ART initiation	< 50 cells/μL from baseline at 1 year after ART initiation	(104)
	> 50 cells/μL from baseline at 1 year after ART initiation	< 50 cells/μL from baseline at 1 year after ART initiation	(105)
	> 50 cells/μL from baseline at 3-9 months after ART initiation	< 50 cells/μL from baseline at 3-9 months after ART initiation	(106–108)
	> 30% from baseline at 1 year after ART initiation	< 20% from baseline at 1 year after ART initiation	(109)
Absolute CD4+ T-cell count and/or CD4+ T-cell count increase	Total count of CD4+ T-cell > 350 cells/μL and/or increase in CD4+ T-cell count > 100 cells/μL from baseline at 48 weeks after ART	Total count of CD4+ T-cell < 350 cells/μL and/or increase in CD4+ T-cell count < 100 cells/μL from baseline at 48 weeks after ART initiation	(110)
	–	Total count of CD4+ T-cell < 350 cells/μL and/or increase in CD4+ T cell count < 50 cells/μL from baseline at 1 year after ART initiation	(111)
	Total count of CD4+ T-cell > 350 cells/μL and/or increase in CD4+ T-cell count > 30% from baseline at 1 year after ART initiation	Total count of CD4+ T-cell < 350 cells/μL and/or increase in CD4+ T-cell count < 30% from baseline at 1 year after ART initiation	(112)
	–	Total count of CD4+ T-cell < 200 cells/μL and/or increase in CD4+ T cell count < 30% from baseline at 1 year after ART initiation	(113, 114)
	Total count of CD4+ T-cell > 200 cells/μL and/or increase in CD4+ T-cell count > 25% from baseline at 1-2 years after ART initiation	Total count of CD4+ T-cell ≤ 200 cells/μL and/or increase in CD4+ T-cell count ≤ 25% from baseline at 1-2 years after ART initiation	(115)
	Total count of CD4+ T-cell > 200 cells/μL and/or increase in CD4+ T-cell count > 20% from baseline at 1-2 years after ART initiation	Total count of CD4+ T-cell < 200 cells/μL and/or increase in CD4+ T-cell count < 20% from baseline at 1-2 years after ART initiation	(116, 117)
CD4/CD8 ratio	≥ 1 at 24 weeks after ART initiation	< 1 at 24 weeks after ART initiation	(118)
Absolute CD4+ T-cell count and CD4/CD8 ratio	Total CD4 + T-cell count > 900 cells/μL from baseline and CD4/CD8 ratio > 1 at 8 years after ART initiation	Total CD4 + T-cell count < 500 cells/μL from baseline and CD4/CD8 ratio < 1 at 8 years after ART initiation	(119)

Initially, it is important to note that some classifications were excluded from the study due to discrepancies in the threshold for defining an undetectable viral load (**Supplementary Material**). In the compiled data, these thresholds ​​ranged from <20 to <1000 copies/mL. Plasma viral load is a critical factor influencing immune reconstitution, since elevated viremia is associated with increased levels of chronic inflammation and immune activation, which negatively affect CD4+ T-cell recovery (7, 27). Therefore, comparing PLHIV with significantly different viral loads is not meaningful. Consequently, this study only included classifications that considered an undetectable viral load <50 copies/mL, in alignment with WHO recommendations (16).

Another important point to highlight is that some studies provided classification only for INRs, while the IR status was not as clearly defined (84, 85, 97, 111). In our view, this circumstance leads to an insufficiently clear classification of IRs, resulting in doubts and questions about immune reconstitution in this group. In this context, the classification adopted by the WHO is noteworthy. The WHO currently characterizes INRs as PLHIV with an absolute CD4+ T-cell count of 250 cells/µL following clinical failure or a persistent count of 100 cells/µL after six months of effective treatment (16). However, the absence of a precise definition for IR within this classification leaves uncertainties regarding what constitutes adequate immune reconstitution, which is essential to provide benefits for PLHIV. We believe that having a clear and objective definition of both groups enhances fidelity and reproducibility in studies on immunological recovery, as well as having a real impact on the prognosis of the HIV infection. Rather than merely suggesting a definition, it is increasingly important to determine what would constitute the ideal immune response for PLHIV in order to achieve a quality of life comparable to that of HIV-negative individuals.

Regarding other classifications, as mentioned previously, various authors use different criteria to characterize immune nonresponse. The most used criterion across studies is the absolute CD4+ T-cell count. However, it is important to emphasize that even within this criterion, there are discrepancies, particularly concerning the threshold of CD4+ T-cells used to classify the INR group. In addition to the CD4+ T-cell count recommended by the WHO (16), other thresholds have been suggested over the years (<200, <350, <400, <500). While all studies provide justifications for their chosen counts and classifications, it is important to discuss some of these points.

Some studies recommend an absolute CD4+ T-cell count of less than 100 cells/µL or 200 cells/µL to classify an INR (see **Table 1**) (7, 17, 95, 96). When a patient exhibits a CD4+ T-cell count below 200 cells/µL, he is in the AIDS stage, the most advanced phase of HIV infection (121). Additionally, when a PLHIV reaches a CD4+ T-cell count < 100 cells/µL, there is an observed increase in mortality rates, even if the patient maintains viral suppression. At this point, the patient has reached the most severe level of immunodepression (122, 123). For instance, according to the WHO classification, an PLHIV with 100 to 250 cells/µL, without clinical complications, is not classified as having immunological nonresponse (16). Nevertheless, as previously mentioned, within this CD4+ T-cell range, PLHIV are at a significantly higher risk of acquiring a wide variety of coinfections compared to PLHIV with higher CD4+ T-cell counts (33). In addition, there are also PLHIV with CD4+ T-cell counts above 200 cells/µL who, despite not experiencing HIV-related complications, may face challenges in immunological recovery and should also be considered as INRs (53). Therefore, using only an absolute CD4+ T-cell count threshold of <100 cells/µL or <200 cells/µL may not be optimal for representing immunological nonresponse. It is crucial to address the following question: “What CD4+ T-cell count corresponds to a satisfactory immune reconstitution in these PLHIV?”

Several authors have identified 500 cells/µL as the absolute minimum CD4+ T-cell count necessary to indicate satisfactory immunological recovery in ART-treated PLHIV (30, 37, 124). Any count below this threshold increases the individual’s susceptibility to opportunistic infections and neoplasms (33). The same concern applies to the other counts mentioned, as they fall below the desired threshold for classifying a patient as IR. Therefore, a CD4+ T-cell count of ≥500 cells/µL is considered a strong indicator of a satisfactory immunological recovery, since at this stage PLHIV and HIV-negative individuals are closely susceptible to similar types of infections (33, 125).

In addition, various classifications consider only the absolute CD4+ T-cell count (see **Table 1**) (62, 70, 71). Although this remains the most used criterion, it is challenging to define immune reconstitution without accounting for the gain in cells during ART. A significant proportion of existing classifications do not include CD4+ T-cell count increase, potentially overlooking a critical aspect of immunological recovery, since this factor directly reflects the mechanisms responsible for CD4+ T-cell reconstitution (7). Thus, disregarding cell gain throughout ART could lead to misinterpretations in the classification process, especially when the increase in CD4+ T-cell count from the initiation of ART to the point of classification is not considered.

For example, by not considering CD4+ T-cell gain, the WHO’s criteria (16) may classify a PLHIV with a CD4+ T-cell count of 130 cells/µL and no clinical complications after 6 months of effective treatment as not exhibiting immunological nonresponse. However, if this patient started ART with 100 cells/µL and gained only 30 cells/µL cells after 6 months, this would not indicate satisfactory immune reconstitution, given that a PLHIV with virological success is expected to gain 20-30 cells/µL per month during this period (**Figure 1A**). In the classification recommended by the WHO, this aspect is not thoroughly assessed, which might occasionally lead to individuals at the AIDS stage, already experiencing significant immunodepression, being classified inaccurately. Thus, we suggest that CD4+ T-cell gain should be strongly considered alongside absolute cell count when differentiating between INR and IR.

Recently, another criterion with significant potential for immunological classification has emerged: the CD4/CD8 ratio. A low CD4/CD8 ratio in ART-treated PLHIV indicates exacerbated immune activation and an elevated risk of morbidity and mortality (126). However, the CD4/CD8 ratio also serves as an indicator of inflammation and other age-related immunological changes, such as arterial stiffness and sarcopenia (127, 128). This suggests that other factors, particularly age, can significantly influence this criterion without necessarily reflecting the process of immune reconstitution, especially when evaluated in isolation (126). Furthermore, following virological suppression, absolute CD4+ T-cell counts increases more slowly than CD8+ T-cell count decrease, which remains significantly elevated for an extended period, resulting in persistently reduced CD4/CD8 ratios even after an increase in CD4+ T-cell count (129).

In addition to determining which criterion to use, there are also divergences regarding the timeframes required to assess immune reconstitution, with suggested durations ranging from six months to seven years (56, 64, 100, 130). However, when evaluating the profile of a patient’s immune reconstitution under ART, it is evident that the most substantial cell gain occurs within the first two years of treatment (**Figure 1A**). After this period, the increase in CD4+ T-cells may reach a plateau (7, 26). Although a gradual rise in CD4+ T-cell count continues over the first seven years of ART, the increase is minimal and unlikely to be decisive in distinguishing between IR and INR (131). This highlights the importance of establishing classifications that incorporate a clearly defined time frame.

The classification proposed by Rb-Silva (132), used two distinct criteria to define IR and INR groups. The choice of criterion depends on the initial CD4+ T-cell count at the initiation of ART, with a specific cutoff of <200 cells/µL. A gain of <50 CD4+ T-cells after a short period of ART (e.g., 6 months) identifies the patient as a potential INR when evaluated over time. Alternatively, an absolute count of <350 cells/µL after a prolonged period of ART (≥24 months) defines PLHIV as INR, as they have likely reached their maximum potential for immunological recovery with persistently low CD4+ T-cell levels. Providing a specific time range for classifying immunological status is essential, as leaving the classification period undefined overlooks that the mechanisms driving CD4+ T-cell increases and the number of cells recovered vary across these periods. Moreover, applying of two different criteria introduces variability to the classification process, hindering standardization and affecting the reproducibility of studies.

It is also important to note that some studies have gaps in group classification, such as defining IR as CD4+ T-cell count ≥500 cells/µL and INR as ≤200 cells/µL (30). In our analysis, this omission leads to significant shortcomings, as it overlooks a substantial subset of PLHIV who do not fit into these predefined groups. To address this issue, some classifications, like that suggested by Cenderello (37), introduce a new group: the partial responders. This group includes PLHIV with a CD4+ T-cell count between 200 and 500 cells/µL within a period of 18 to 36 months, falling between the IR and INR classifications. The introduction of this new group allows for the classification of PLHIV who were uncategorized as either IR or INR. However, proposing another category may hinder the classification process, making it more challenging to reproduce and effectively apply. Furthermore, this classification shares the same limitation as the previously mentioned one: It lacks a precise time frame for patient classification.

All these various divergences in classification complicate both the understanding of immunological recovery and progress in research on this condition. This highlights the need for a classification system that accounts for all relevant aspects and accurately represents patient status. Our proposed system addresses this challenge by consolidating PLHIV into two comprehensive categories, ensuring that all individuals are properly classified.

## Proposed immunological classification

**Figure 4:**
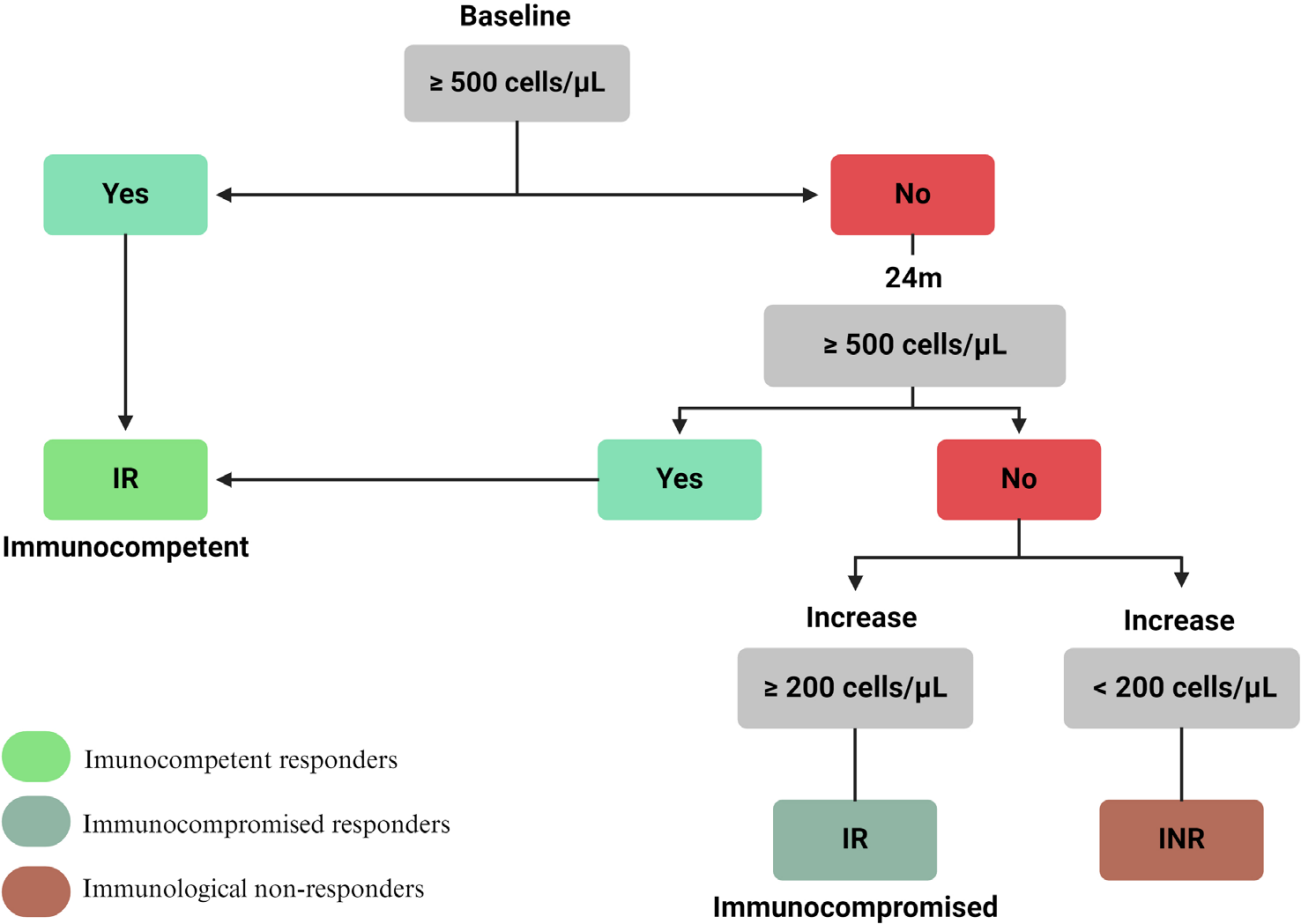
Proposed classification for immunological recovery in ART-treated PLHIV. The initial assessment involves measuring the CD4+ T-cell count at baseline. PLHIV with ≥ 500 cells/µL and maintaining after 24 months are classified as immunological responders (IR) and are considered to have an immunocompetent system. Those with a lower CD4+ T-cells count at ART initiation undergo reassessment after 24 months. If the PLHIV achieve a count of ≥ 500 cells/µL within this period, they are also classified as IR. Those who remain below this threshold are further evaluated based on their CD4+ T-cell increase: a gain of ≥ 200 cells/µL results in an IR classification albeit with an immunocompromised system, while a gain of <200 cells/µL classified them as immunological non-responders (INR).

In light of the above, we propose a classification that combines the two most representative criteria: the absolute CD4+ T- cell count and the gain in these cells over time (**Figure 4**).

The classification is initially based on the absolute baseline CD4+ T-cell count for PLHIV who have achieved virological suppression (<50 RNA copies/mL). PLHIV initiating ART with a baseline CD4+ T-cell count of ≥500 cells/µL and maintained after 24 months will be defined as IR. Conversely, PLHIV with counts below this level will have their absolute CD4+ T-cell count evaluated after 24 months of ART. If, after this period, their CD4+ T-cell count achieves ≥500 cells/µL, they will also be classified as IR, signifying immunocompetence in both situations (**Figure 4**). Essentially, their immune system can effectively develop response and control infections caused by other pathogens (133). PLHIV not reaching this threshold will be further analyzed based on cell gain over the same period. Those gaining ≥200 CD4+ T-cells/µL will be classified as IR but immunocompromised, while those gaining <200 CD4+ T-cells/µL will be classified as INR (**Figure 4**).

As mentioned earlier, only PLHIV with an absolute CD4+ T-cell count of ≥500 cells/µL are immunologically equivalent to HIV-negative individuals. Even though PLHIV who gain ≥200 cells/µL after 24 months are considered IR, it is important to highlight that if they do not achieve a CD4+ T-cell count of ≥500 cells/µL, they remain immunocompromised, with a reduced capacity to combat and control infection by other pathogens (33). These PLHIV exhibit higher morbidity rates compared to those who reach the ≥500 cells/µL threshold (37, 134). Given the scarcity of classifications that incorporate this factor, assessing immunocompromised status is essential when distinguishing between IR and INR, as it directly impacts immune response competence and the PLHIV’s quality of life.

## Conclusion

The lack of consensus among authors regarding criteria for immunological classification in ART-treated PLHIV significantly impacts advancements in this area. Establishing an objective and precise classification to define IR and INR PLHIV is crucial for the scientific community, as it not only addresses a critical gap in current research but also has implications for clinical practice. Additionally, it can be employed for risk stratification in the development of both AIDS-related and non-AIDS-related complications, as well as for providing a more effective prognosis of the immune response to treatment. Furthermore, our aim extends beyond defining immunological nonresponse; we seek to delineate what constitutes satisfactory immune reconstitution, ultimately aiming to enhance the quality of life of PLHIV. We believe that a simplified and standardized classification system will facilitate reproducibility across studies, preventing classification gaps, and ensure that all PLHIV are categorized into one of the defined groups. Moreover, given its objectivity and practicality, the classification has the potential to be incorporated into clinical practice, aiding in the management and monitoring of PLHIV. Thus, this study proposes a comprehensive classification that integrates relevant factors in immune reconstitution assessment, laying a foundation for future studies on immunological nonresponse in ART-treated PLHIV. In addition, we encouraged future studies to directly compare the performance of our classification system with existing ones, particularly in predicting clinical outcomes and guiding therapeutic decisions.
